# USING TECHNOLOGY TO FACILITATE SUPPORTIVE SERVICES FOR HOSPICE FAMILY CAREGIVERS: FINDINGS FROM THE PISCES TRIAL

**DOI:** 10.1093/geroni/igz038.719

**Published:** 2019-11-08

**Authors:** George Demiris

**Affiliations:** University of Pennsylvania, Philadelphia, Pennsylvania, United States

## Abstract

Family caregivers of hospice patients play an essential role in the delivery of care but have multiple needs during this often stressful time. Cost-effective interventions supporting caregivers are greatly needed. We conducted a four-year randomized clinical trial of a problem solving therapy intervention called PISCES to support hospice caregivers. We recruited 514 caregivers (75% female, mean age 60.3 years) who were randomly assigned to either a control group (usual care) or a face to face group (where the intervention was delivered in three in-person sessions) or a telehealth group (where the intervention was delivered using technology). While the intervention was effective in reducing caregiver anxiety and improving overall quality of life, the intervention was found more effective in the face-to-face group than the telehealth group. We discuss challenges with the technology use and recommendations for the design of future telehealth systems targeting older adults in the hospice setting.

